# Proactive approach to recreation and efficiency of recovery in flexible work arrangements

**DOI:** 10.1192/j.eurpsy.2022.1777

**Published:** 2022-09-01

**Authors:** A. Kuznetsova, M. Luzyanina, M. Titova

**Affiliations:** 1 Lomonosov Moscow State University, Department Of Psychology, Moscow, Russian Federation; 2 The Moscow City University of Management (MSUU) of the Moscow Government, Center For Personnel Diagnostics And Personnel Development, Москва, Russian Federation

**Keywords:** flexible work arrangements, recreation planning, proactive/reactive approach to recreation, recovery

## Abstract

**Introduction:**

Flexible work arrangements promote not only acceptable and convenient work modes; for many professionals flexible work leads to increase in workload and in working time (Rubery et al., 2016; Thompson et al., 2015). As the result, lack of recreation time could be named as a direct consequence of high workload (Pang, 2017). The key problem is the investigation of attitudes towards recreation and recovery: are professionals more reactive or proactive in their recreation planning, and do they recover well?

**Objectives:**

The aim of the research: to reveal (1) typical types of recreation planning for professionals with high level of work flexibility and (2) recovery efficiency level.

**Methods:**

The research was conducted in representatives of various professions, who work in flexible work arrangements (n=378). The diagnostic set included inventories for assessment of recreation planning type (Luzyanina, Kuznetsova, 2014) and recovery efficiency (Leonova, 2019).

**Results:**

Two types of recreation planning have been found: proactive (26% of respondents) and reactive (74%). For the reactive approach lack of targeted strategies of recreation planning has been found. Proactive approach is characterized by tracking signs of resources decrease and advance planning of work breaks. There are differences in recovery efficiency (p<0,001) in proactive and reactive professionals: non-efficient recovery is typical for the majority of professionals with the reactive type to recreation planning.

**Conclusions:**

The detailed analysis of proactive/reactive approaches manifestations and peculiarities of recreation planning could help to predict not only the recovery level, but the mechanisms of advanced self-regulation, adequate to high work flexibility.

**Disclosure:**

No significant relationships.

